# Perfluorodecyltrichlorosilane-based seed-layer for improved chemical vapour deposition of ultrathin hafnium dioxide films on graphene

**DOI:** 10.1038/srep29223

**Published:** 2016-07-06

**Authors:** Julia Kitzmann, Alexander Göritz, Mirko Fraschke, Mindaugas Lukosius, Christian Wenger, Andre Wolff, Grzegorz Lupina

**Affiliations:** 1 IHP GmbH–Leibniz institute for innovative microelectronics, Im Technologiepark 25, 15236 Frankfurt (Oder), Germany

## Abstract

We investigate the use of perfluorodecyltrichlorosilane-based self-assembled monolayer as seeding layer for chemical vapour deposition of HfO_2_ on large area CVD graphene. The deposition and evolution of the FDTS-based seed layer is investigated by X-ray photoelectron spectroscopy, Auger electron spectroscopy, and transmission electron microscopy. Crystalline quality of graphene transferred from Cu is monitored during formation of the seed layer as well as the HfO_2_ growth using Raman spectroscopy. We demonstrate that FDTS-based seed layer significantly improves nucleation of HfO_2_ layers so that graphene can be coated in a conformal way with HfO_2_ layers as thin as 10 nm. Proof-of-concept experiments on 200 mm wafers presented here validate applicability of the proposed approach to wafer scale graphene device fabrication.

Due to its unique properties graphene is a potential material for use in next-generation micro- and nanoelectronics^1^. For these envisioned applications including field-effect transistors^2^,^3^,^4^, graphene base transistors^5^,^6^,^7^ and optoelectronic devices^8^ thin conformal dielectric and semiconductor layers have to be grown on graphene. The direct growth of dielectrics on graphene was reported with several methods such as chemical vapour deposition (CVD)^9^, plasma-enhanced CVD^10^ and atomic layer deposition (ALD)^11^, but the deposition of conformal insulating layers usually requires formation of a seed layer due to the lack of dangling bonds on a defect-free surface of graphene^12^. Different seeding layers improving nucleation were investigated including low-*k* polymers^13^,^14^, thin metal films^15^,^16^, substrate induced seeding^17^ or a pre-treatment with NF_3_^18^. These approaches significantly improve nucleation in ALD and CVD processes, however, they increase process complexity and are often not compatible with Si technology standards.

In this work we investigate a pre-treatment of graphene with perfluorodecyltrichlorosilane (FDTS) prior to HfO_2_ deposition on blanket Silicon 8 inch wafers as well as on pre-structured TiN films. We adapt an established process used for surface energy modification in the fabrication of micro-electro-mechanical systems (MEMS) structures and their cavities^19^. We demonstrate that the FDTS self-assembled monolayer (SAM) significantly improves nucleation of HfO_2_ on graphene. Wafer scale compatibility and maturity of the proposed pre-treatment can enable fast adoption for the fabrication of graphene-based devices on large-diameter wafer scale.

## Results and Discussion

**Figure 1:**
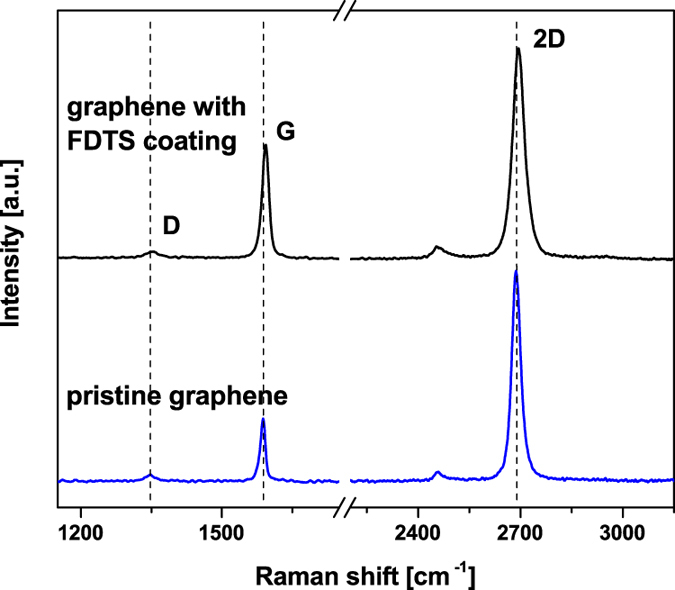
Comparison of Raman spectra from graphene taken before and after FDTS coating.

The representative Raman spectra of a graphene layer transferred to 300 nm SiO_2_/Si(100) wafers before and after FDTS coating are shown in Fig. 1. A strong G and 2D bands and a small D band confirm a high quality of the as-transferred layer^20^,^21^. After coating with FDTS the D band remains barely visible. G and 2D bands are narrow (FWHM(G): 14 cm^−1^, FWHM(2D): 36 cm^−1^) proving that the FDTS coating process does not negatively affect the crystalline quality of graphene sheet. The observed red shift of about 8 cm^−1^ after SAM formation may be associated with a process induced electrostatic doping of graphene^22^,^23^.

**Figure 2:**
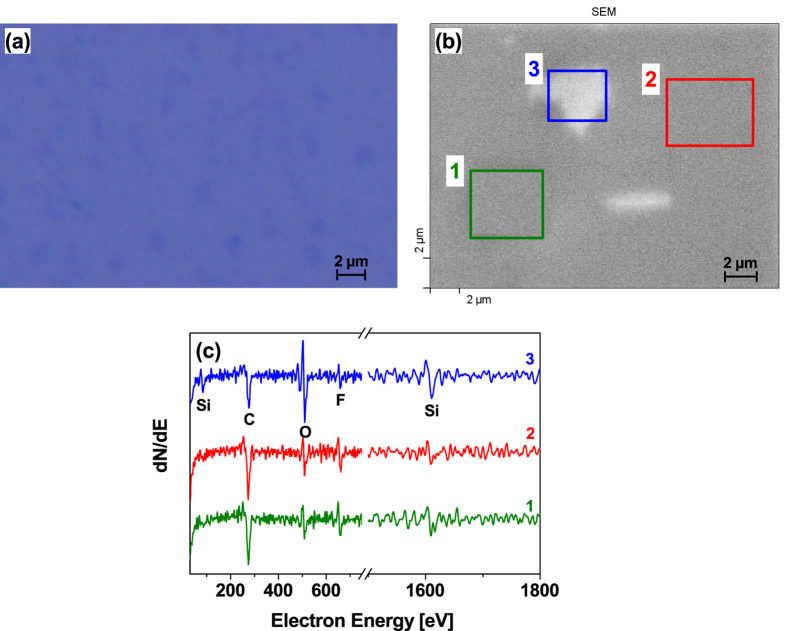
Inspection of the transferred graphene layer after FDTS coating process. (**a**) Optical microscope image. (**b**) SEM micrograph and corresponding Auger electron spectra (**c**) acquired from three characteristic areas 1, 2 and 3 marked with green, red and blue rectangles.

**Figure 3:**
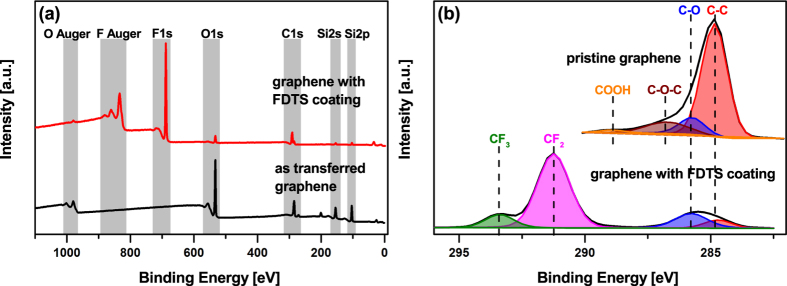
Full survey XPS spectra (a) and high-resolution spectra of C1s peak (b) of pristine transferred graphene layers and of FDTS coated graphene.

The optical microscope image of a transferred graphene layer after the FDTS deposition process is shown Fig. 2a. Caused by the growth and transfer process, a small number of multi-layer islands and few cracks and holes are visible in the microscopic pattern. However, the FDTS based self-assembled monolayer is not visible by optical microscope imaging. In order to demonstrate the successful coating of graphene surface with FDTS, combined secondary electron microscopy (SEM) and Auger electron spectroscopy (AES) measurements were performed, as illustrated in Fig. 2b,c, respectively. The bright region in Fig. 2b (blue rectangle) is associated with a hole in the graphene layer whereas the remaining area is covered with graphene. As expected, the corresponding AES spectrum recorded in region 3 (Fig. 2c) shows intense Si and O signals originating from the SiO_2_ substrate and weaker C signal due to the absence of graphene. The additional scans performed on the remaining area (regions 1 and 2) show reduced Si and O signals and an intense C peak due to the closed graphene layer. A fluorine signal of similar intensity is detected in all investigated regions proving that the FDTS SAM was coated homogeneously on the substrate surface. More detailed chemical analysis of the SiO_2_/graphene/FDTS interfaces was performed by X-ray photoelectron spectroscopy (XPS), as shown in Fig. 3.

Overview XPS spectra of pristine transferred graphene layers and of the processed samples were performed to monitor the chemical induced changes by FDTS coating, as illustrated in Fig. 3a. The chemical differences are clearly visible. In the spectra of the pristine graphene layer no F peak was monitored, whereby the O1s peak and the Si peaks are visible due to the SiO_2_ substrate which can be detected through the graphene layer. After the FDTS coating process, a clear F1s appears and the O1s peak and Si 2s and 2p peaks are attenuated. Based on these XPS results, we can also conclude, that no additional elements are incorporated during the FDTS deposition into the graphene layer (Fig. 3a).

A detailed compositional analysis of the C1s peak is presented in Fig. 3b. The pristine graphene (Fig. 3b) shows the typically XPS spectrum of C1s with the presence of a C-C peak around 284.8 eV which is characteristic for sp^2^ bonded carbon atoms^24^,^25^. Due to the hydrocarbon contamination by air exposure, an additional C-O peak is recorded and carbonyl and carboxyl groups caused by grain boundary defects are visible^24^,^25^. The spectrum of the C1s signal after FDTS coating is completely different. Now, the typical XPS spectrum of FDTS SAM is recorded. The main peak from graphene (284.8 eV) is significantly reduced and new peaks are detected at larger binding energies. These peaks are attributed to carbon-fluorine bonds: CF_2_ functional backbone group and CF_3_ tail group at energy values of 291 eV and 293 eV respectively^26^,^27^,^28^,^29^ (see curve fitting in Fig. 3b). The oxidized species C-O are detected at a binding energy of around 286 eV. The intense CF_2_ signal at 297 eV is caused by the fluorine-chains which were deposited on the graphene surface.

**Figure 4:**
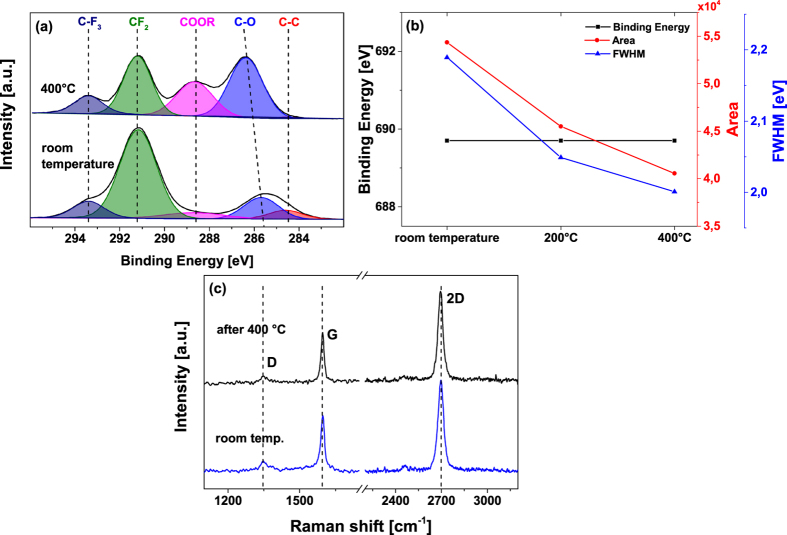
High-resolution XPS spectra of the C1s signal (a,b) and Raman spectra (c) of the FDTS coated graphene layer obtained at different temperatures. (**a**) Analysis of the C1s signal at room temperature and after the annealing at 400 °C. (**b**) Extracted binding energies, peak area and full width at half maxima (FWHM) of the F1s signal as function of annealing temperature. (**c**) Raman spectra of the graphene sample before and after annealing.

To determine the impact of temperature to the stability of the FDTS based seed layer, a study at different temperatures was performed in ultra-high vacuum. The XPS spectra were recorded *in situ*. The sample was firstly investigated at room temperature; afterwards the first annealing step was performed at 200 °C for 5 minutes. Since the HfO_2_ deposition process after seed layer formation is performed at 400 °C, the second annealing step was set also to 400 °C for 5 minutes. As illustrated in Fig. 4, XPS measurements were directly performed *in-situ* in order to record the chemical changes of the coated FDTS layer.

The intensity of the aromatic C-C signal is reduced after the annealing at 400 °C, as illustrated in Fig. 4a. Also the CF_2_ signal is reduced; while the intensity of the oxidized carbon species (C-O) rises with temperature. As to be seen in the spectra, the characteristic oxidized carbon species (COOR) signals are also observed at a binding energy of 288 eV^28^,^29^. These changes in the C1s spectrum are caused by the cracking of the long fluorine chains in FDTS at 400 °C. Due to this cracking, oxygen from SiO_2_/Si substrate is adsorbed, resulting in an increase of the signal of oxidized carbon species C-O and COOR and a peak shift of the C-O component^28^,^29^. In order to study the chemical changes more detailed, the F1s signal is also evaluated at the annealing temperature of 200 °C. The results of the F1s signal analysis are shown in Fig. 4b. It is obvious that the binding energy does not shift with increasing temperature, but the intensity of the signal decreases with raising temperature. As illustrated in Fig. 4b the peak area as well as the full width at half maxima decrease with raising temperature, meaning that the desorption of fluorine species starts already at 200 °C and is even more pronounced at 400 °C.

As the decomposed FDTS chains could interact with graphene, a Raman analysis was performed after the annealing procedure in order to investigate the quality of the graphene layer. As illustrated in Fig. 4c, no destruction of the graphene layer occurs when the FDTS layer is decomposed by temperature.

The following conclusions can be drawn from the discussions above: The FDTS seed layer covers graphene homogenously at room temperature and this process does not affect the crystalline quality of graphene. During thermal treatment, long fluorine chains partially decompose and some fluorine species desorb from the surface as it is evidenced by reduced F1s signal. After annealing the high crystalline quality of graphene remains unaffected as proved by Raman spectroscopy investigations.

The next section is dedicated to the growth of HfO_2_ by CVD on pretreated graphene layers. As demonstrated by Lukosius *et al*.^30^, the direct growth of HfO_2_ on transferred graphene is feasible without any initial pretreatment of the graphene surface with the restriction, that HfO_2_ films have to be thicker than 20 nm to be uniform and closed. Due to nucleation sites created by the FDTS based seed layer, the minimum HfO_2_ film thickness should be drastically reduced. In the first step, we chose a thickness of nominally 10 nm HfO_2_ to test the influence of the FDTS nucleation capability. Two graphene samples were transferred to pre-structured 75 nm TiN/Si substrates, which will be used for ongoing electrical characterization. After the transfer process one sample was coated with FDTS, while the other sample was left pristine. Finally, 10 nm HfO_2_ were deposited by CVD onto both samples.

**Figure 5:**
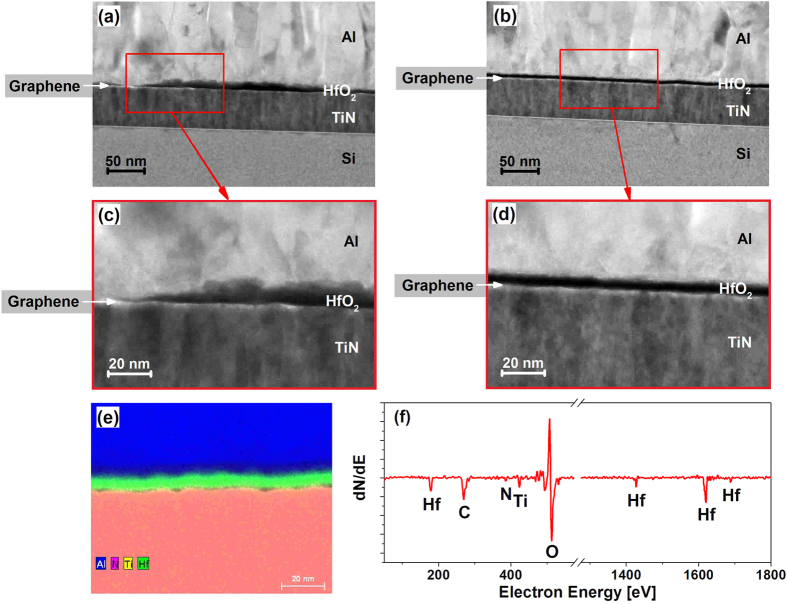
HR TEM images of samples after nominally 10 nm HfO_2_ deposition (a–d). (**a**) Without FDTS coating and (**b**) with FDTS coating, (**c**,**d**) show magnification of the selected regions, marked with red rectangles. **(e)** EDX-spectrum and (**f**) AES-analysis before Al-coating.

In order to investigate the quality of the HfO_2_ layer, transmission electron microscopy (TEM) was applied. The samples were firstly coated with an Al-layer to protect the sample and then a lamella was prepared. According to the high resolution TEM images, there is a huge difference between the two samples, as illustrated in Fig. 5. While the sample without FDTS coating (Fig. 5a,c) shows a non-uniform and very rough HfO_2_ layer, the sample with FDTS coating (Fig. 5b,d) shows a closed, uniform and smooth HfO_2_ layer. The FDTS coating clearly optimizes the growth of thin layers of HfO_2_ on graphene. To proof the chemical composition of the deposited HfO_2_ layer an EDX-analysis (Fig. 5e) and an Auger electron spectroscopy (Fig. 5f) were done. In the AES-spectrum no Al peak can be seen, because the measurement was done before coating the sample with Aluminium for TEM. The Auger analysis was measured before coating the sample with aluminium. Both measurements show a thin HfO_2_ layer, grown on top of the sample.

## Conclusions

The pre-treatment, done by a modified FDTS process can be used to improve the growth of high-*k* dielectric layers on graphene with an excellent quality. We have demonstrated, that the minimum thickness of closed and uniform grown of HfO_2_ by CVD on pre-treated FDTS graphene layers can be scaled down to 10 nm. The results implicate that the pre-treatment with FDTS provide a new route to deposit thin conformal layers of HfO_2_ dielectrics on graphene surfaces up to 200mm wafer size.

## Methods

### Sample preparation

Commercially available graphene was transferred from Cu growth substrates by detaching graphene from Cu foils with poly(methyl methacrylate) (PMMA) assisted wet etching in Ammonium persulfate (80 mg/ml in water)^31^. After etching the PMMA/graphene stack was rinsed in fresh distilled water for several times to remove etchant solution. The stack with a size of 1 × 1 cm^2^ was then transferred to target substrate and immersed in acetone and finally rinsed in isopropyl alcohol (IPA) to remove the polymer. Target substrates in this study were 200 mm Si(100) wafers covered with either 300 nm thermal SiO_2_ or 75 nm CVD TiN layers.

### FDTS coating and HfO_2_ deposition

The FDTS (CF_3_(CF_2_)_7_(CH_2_)_2_SiCl_3_) coating of graphene was carried out with a 200 mm AURIX tool (memsstar Limited, Livingston, UK). The standard FDTS coating process used in MEMS fabrication is performed at substrate temperatures of 30 °C and is divided into two parts: the first part cleans and activates the surface by exposure of the wafer to O_2_ plasma, the second part provides the N_2_ bubbled exposure to FDTS and DI water at a process pressure of 40 Torr and for a duration of 300 s. The flow rate of the two carrier gases are 20 sccm for FDTS and 100 sccm for DI water. As O_2_ plasma readily introduces defects in the crystalline lattice of graphene, this step was omitted in the process flow.

HfO_2_ growth was performed in an atomic vapor deposition tool with Hf(NMeEt)_4_ precursor and oxygen as the reactive gas^9^. The substrate temperature during HfO_2_ deposition was set to 400 °C for a duration of 3 min. The deposited layers are polycrystalline. More details about the depositions of HfO_2_ by CVD have been reported elsewhere^32^.

### Characterization

A Renishaw InVia micro-Raman spectrometer equipped with a 514 nm wavelength laser, an 1800 lines/mm grating and 50x objective was used to acquire Raman spectra. XPS measurements were performed using PHI 5000 Versa Probe II (ULVAC-PHI) with monochromatic Al-Kα irradiation. The chamber pressure was ~3 × 10^−8^ Torr and the spectra were measured with X-ray beam size of 100 × 100 μm. A PHI 670 Auger Nanoprobe (Perkin Elmer) was used for Auger electron spectroscopy. The spectra were measured with a beam voltage of 10 keV. Structural investigations and EDX-measurements were done by transmission electron microscopy using a FEI Tecnai Osiris instrument operated at 200 kV.

## Additional Information

**How to cite this article**: Kitzmann, J. *et al*. Perfluorodecyltrichlorosilane-based seed-layer for improved chemical vapour deposition of ultrathin hafnium dioxide films on graphene. *Sci. Rep.* **6**, 29223; doi: 10.1038/srep29223 (2016).
